# Do Mirror Glasses Have the Same Effect on Brain Activity as a Mirror Box? Evidence from a Functional Magnetic Resonance Imaging Study with Healthy Subjects

**DOI:** 10.1371/journal.pone.0127694

**Published:** 2015-05-27

**Authors:** Christopher Milde, Mariela Rance, Pinar Kirsch, Jörg Trojan, Xaver Fuchs, Jens Foell, Robin Bekrater-Bodmann, Herta Flor, Martin Diers

**Affiliations:** 1 Department of Cognitive and Clinical Neuroscience, Central Institute of Mental Health, Medical Faculty Mannheim, Heidelberg University, Mannheim, Germany; 2 Biological Psychology, University Koblenz-Landau, Landau, Germany; 3 Department of Psychology, Florida State University, Tallahassee, Florida, United States of America; University of Montreal, CANADA

## Abstract

Since its original proposal, mirror therapy has been established as a successful neurorehabilitative intervention in several neurological disorders to recover motor function or to relieve pain. Mirror therapy seems to operate by reactivating the contralesional representation of the non-mirrored limb in primary motor- and somatosensory cortex. However, mirror boxes have some limitations which prompted the use of additional mirror visual feedback devices. The present study evaluated the utility of mirror glasses compared to a mirror box. We also tested the hypothesis that increased interhemispheric communication between the motor hand areas is the mechanism by which mirror visual feedback recruits the representation of the non-mirrored limb. Therefore, mirror illusion capacity and brain activations were measured in a within-subject design during both mirror visual feedback conditions in counterbalanced order with 20 healthy subjects inside a magnetic resonance imaging scanner. Furthermore, we analyzed task-dependent functional connectivity between motor hand representations using psychophysiological interaction analysis during both mirror tasks. Neither the subjective quality of mirror illusions nor the patterns of functional brain activation differed between the mirror tasks. The sensorimotor representation of the non-mirrored hand was recruited in both mirror tasks. However, a significant increase in interhemispheric connectivity between the hand areas was only observed in the mirror glasses condition, suggesting different mechanisms for the recruitment of the representation of the non-mirrored hand in the two mirror tasks. We conclude that the mirror glasses might be a promising alternative to the mirror box, as they induce similar patterns of brain activation. Moreover, the mirror glasses can be easy applied in therapy and research. We want to emphasize that the neuronal mechanisms for the recruitment of the affected limb representation might differ depending on conceptual differences between MVF devices. However, our findings need to be validated within specific patient groups.

## Introduction

The idea of using altered visual feedback to relieve phantom limb pain by using a mirror box (MB) was originally proposed by Ramachandran et al. [1]. Since then mirror visual feedback (MVF) has been established in the treatment of phantom limb pain [2–4], but also as an important therapeutic tool for functional recovery after a stroke [5–7], physiotherapy after wrist fracture [8], the treatment of complex regional pain syndrome [9,10] or for reinstating body ownership in somatoparaphrenia [11].

The basic idea of MVF is that extended viewing of movements of the unaffected limb visually superimposed on the affected limb by a sagittally placed mirror triggers the perception that the phantom (or affected) limb is moving [12]. Whereas the beneficial effects of MVF have been repeatedly demonstrated, the mechanisms underlying MVF-induced improvements in motor function and pain relief remain unclear [13,14]. There is increasing evidence that a reactivation of the affected limb representation in the sensorimotor strip and accompanying neuroplasticity is an important neuronal correlate of the MVF related neurorehabilitation [13,15,16]. However, it remains unclear how the sensorimotor representation of the non-mirrored (affected) limb becomes functionally recruited because studies examining the functional connectivity between brain areas during MVF are still rare [13,17].

In the MB approach, the (affected) limb is positioned behind a mirror, which is oriented along the observer’s midline so that the visual reflection of a moving (intact) limb visually replaces the hidden (affected) limb. Using a MB in therapy and research is constrained by several technical and conceptual limitations such as size and weight, which reduces the degrees of freedom for possible movements in front of the mirror and constrains its applicability in therapy and in magnetic resonance imaging (MRI) setups [18]. In contrast, mirror glasses (MG) limit the field of view to the visual reflection of the moving (intact) limb which replaces the hidden (affected) limb in the visual field whereby the actually moving limb is visually occluded. This is achieved by covering the eye ipsilateral to the movement and mirroring the visual hemifield to the other eye. It has been proposed that seeing the actual moving hand, in addition to the visual reflection of the moving hand, might be an irrelevant distractor reducing the ability of the subject to stay focused on the reflection of the moving hand [18,19]. Thus MG might have a higher capability of recruiting the motor representation ipsilateral to the moving hand (further referred to as MI_ipsi_) compared to MB by enabling increased spatial attention towards the reflection of the moving (affected) limb [19]. MG deliver a more realistic image of the mirrored limb than virtual reality systems, which has been shown to be an important aspect of perceiving body illusions [20]. Additionally, MG are smaller in size and weight than the MB. Thus MG might be more attractive for healthcare providers and more appropriate in functional MRI (fMRI) paradigms [18]. Compared to other studies, which focused on classical or virtual applications of the MB [15,16,21], this is the first study systematically investigating the subjective quality and associated functional brain activity provided by MG which limit the field of view to the visual reflection of the moving (intact) limb.

To evaluate the efficiency of MG, we examined 20 healthy subjects in a counterbalanced within-subjects design with MVF provided either by MB or MG. We assessed subjective ratings on the intensity and vividness of mirror illusions as well as fMRI data. Due to the putatively distracting effect of seeing the moving hand in addition to the visual reflection of the moving hand, we hypothesized to find higher subjective mirror illusion capacities as well as an increased recruitment of MI_ipsi_ in the MG compared to the MB condition. Moreover, we analyzed task-dependent functional connectivity between both hand areas, as one proposed neural mechanism for the recruitment of the sensorimotor representation corresponding to the hidden (affected) limb [13].

## Methods

### Participants

Twenty healthy subjects (*M* = 31.3 years, *SD* = 7.7 years; 15 females) took part in the study. Participants were right handed as assessed with the Edinburgh Handedness Inventory [22], reported normal or corrected-to-normal vision, had no history of neurological disease and did not use any centrally acting medication such as opiates. We first wanted to evaluate the effects of MG in a group of healthy subjects before using this device in specific patient groups.

### Ethics Statement

The participants gave written informed consent in accordance with the Declaration of Helsinki (2008) prior to participation. The study was approved by the Ethics Committee of the Medical Faculty Mannheim, Heidelberg University (internal reference: 2008-336N- MA).

### Mirror Glasses

**Fig 1:**
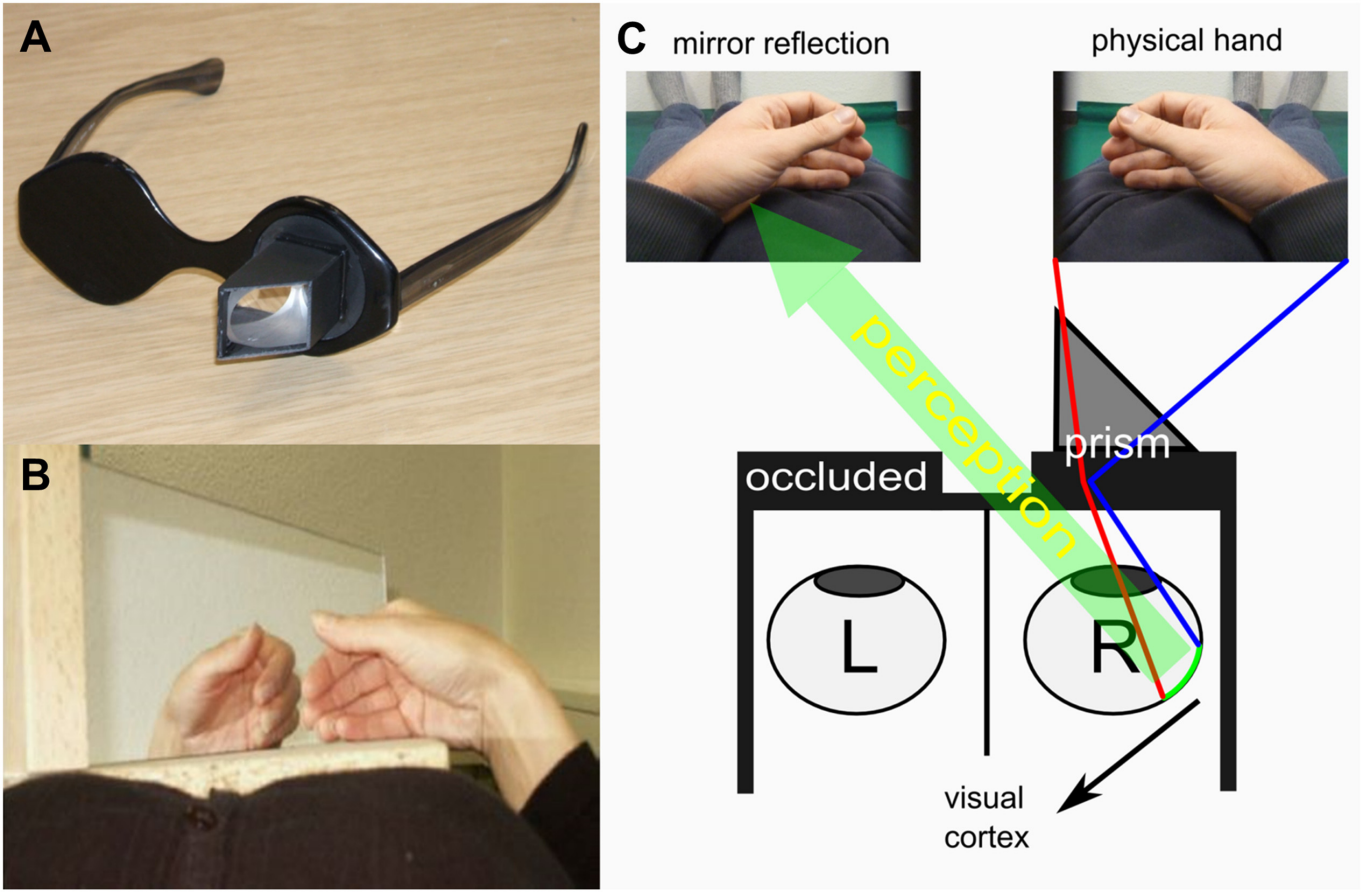
Mirror visual feedback (MVF) devices. (A) Mirror glasses: are usable within an MR environment. The optical path was deflected by a prism, which was a 1.5–1.53 45-90-45 angled glass, Barium crown (BK-7, Abbe 63) with quarter wavelength surface tolerance. (B) Mirror box: was a framed glass mirror (size: 35 by 12 centimetres / 13.8 by 4.7 inches) which was placed on the abdomen of the subject providing view on the executing hand as well as the visual reflection of the hand appearing to move in synchrony. During both conditions view on the mirror reflection of the moving limb was provided by means of an additional mirror attached to the head coil. (C) Illustration of the MVF as provided by the mirror glasses: in contrast to the mirror box the users’ view is limited to the mirror reflection of the moving (physical) hand as opposed to seeing both hands (physical hand and visual reflection of the physical hand). The mirror reflection of the physical hand was seen through on eye by means of a prism leading to a total inversion in the left-right dimension (in our setup the right hand movements were seen through the right eye appearing as left hand movements). Furthermore, mirror glasses provide a much larger field of view, allowing the whole limb to be inverted.

The MG (Scottish Health Innovations Limited, Glasgow, Scotland) can be used within a MRI environment due to the absence of any ferromagnetic components. The MG limit the field of view to the visual reflection of the moving limb by reflecting the field of view to the eye contralateral to the moving limb. In our setup the field of view was restricted to the mirror reflection of the moving right hand (visually appearing as left hand) which was seen through the right eye (Fig 1). In contrast, the MB provides a view of the actual moving limb together with the visual reflection of the moving limb appearing to move in synchrony. Furthermore, the MG has a larger field of view compared to the MB, including the entire half of the body with its natural range of movements (Fig 1).

### Experimental procedure

The participants were tested in a counterbalanced within-subjects design for the two conditions MB and MG inside the scanner. In the MG condition, participants wore MG, during the MB condition a MB was placed on the abdomen of the subject, enabling them to view the mirrored right hand (appearing as left hand) as well as the actual right hand (Fig 1). In both MVF conditions participants were instructed to repeatedly close and open their right hand at a frequency of 1 Hz as paced by an auditory signal presented via earphones. During movement trials participants were instructed to focus on the visual reflection of the moving right hand. Participants kept their left hand immobile and out of view in a comfortable position on their abdomen. During the experiment the participants view was redirected using a mirror attached to the MRI head-coil. This way, they could easily observe the upper half of the body including the actual or illusory limb movements.

### Subjective ratings on mirror illusions

After each MVF condition, the intensity and vividness of mirror illusions were verbally assessed using a seven-point numeric rating scale. The scale ranged from 1 (‘as clear and vivid as a real perceptual experience’) to 7 (‘not at all clear and vivid’) and was modeled after the Questionnaire upon Mental Imagery [23]. The questions have been used in previous studies [15,16,24]. Mirror illusion items were: Did you feel that the movement of the displayed hand belonged to your left hand? (Vividness) How clearly did you feel the movement of your left hand? (Intensity)

### MRI data acquisition

During execution of both MVF tasks, a Siemens 3 T MAGNETOM Trio whole-body scanner (Siemens AG, Erlangen, Germany) was used in combination with a 12-channel radio-frequency head coil to obtain eighty whole-brain T_2_*-weighted gradient-echo echo planar imaging (EPI) volumes with blood related oxygen level-dependent contrast [repetition time (TR) = 3.3 s; echo time (TE) = 45 ms; flip angle (α) = 90°]. Imaging volumes consisted of 40 slices angulated in parallel to the anterior commissure-posterior commissure with a gap of 0.69 mm recorded in ascending order. Each slice had a matrix size of 96 x 96 voxels with an anisotropic voxel-size of 2.3 x 2.3 x 2.9 mm. For each MVF condition, participants were tested in an alternating block design consisting of six blocks of right-hand movements interspersed by seven baseline blocks. Each block consisted of six scans. Both conditions were split into two separate sessions of about five minutes separated by a five-minute break.

Within the same session, a T_1_-weighted scan (160 contiguous slice, matrix size 240 x 256 voxels, voxel-size = 1 x 1 x 1.1 mm) was conducted to collect a high-resolution structural volume for anatomical reference. The magnetization-prepared rapid acquisition gradient-echo sequence was employed with TR = 2.3 s, TE = 2.98 ms, and α = 9°.

### Statistical analysis of fMRI data

The MRI data were analyzed using tools from FMRIB's Software Library (FSL) version 5.02 [25]. The first five EPI volumes were discarded prior to preprocessing to account for T1-equilibration effects. Prior to statistical estimation, the following preprocessing steps were subjected: Intramodal motion correction using MCFLIRT [26], spatial smoothing using an isotropic Gaussian kernel of 5 mm (full width at half maximum), mean-based intensity normalization of all volumes, and high-pass temporal filtering (σ = 100 s). Registration was performed in 2-steps: EPI volumes were first spatially realigned to the high-resolution T1-weighted volume, where non-brain structures were removed using Brain Extraction Tool (BET) [27]. EPI images were then registered to the standard MNI152 space (Montreal Neurological Institute, Montreal, Canada) using non-affine FNIRT-registration [28] with a warp-resolution of 8 mm. Time-series statistical analysis was carried out using the prewhitening tool FMRIB’s Improved Linear Model (FILM) with local autocorrelation correction.

Functional MRI statistical analysis was carried out using fMRI Expert Analysis Tool (FEAT) [25]. Data from each subject and session (MG; MB) were analyzed at a first-level of analysis. Trials of performing the MVF tasks were used as one factor of interest and convolved with a double-gamma function to model the hemodynamic response function and were entered as a predictor into a general linear model. To account for movement-related artifacts in the signal, the six rigid-body movement parameters were additionally included as nuisance regressors in the design matrix. Brain areas were identified based on the FSL Harvard-Oxford Atlas [29]

Inter-session (MG > MB and MB < MG) and group analyses were carried out using FMRIB’s Local Analysis of Mixed Effects (FLAME) [30]. Areas of significant fMRI activations associated with both MVF conditions were calculated by entering the first-level (sessions) statistics into a second-level mixed-effects group analysis. To compare brain activations between both MVF conditions, we contrasted both MVF sessions (MG > MB and MG < MB) for each subject within a fixed-effects analysis, which was subsequently entered into a third-level mixed-effects group analysis. Areas of significant fMRI-response were determined using clusters identified by a *z* > 3.0 threshold and a corrected cluster threshold of p = 0.05 assuming a Gaussian random field for the *z*-statistics.

#### Psychophysiological interaction analysis (PPI)

Psychophysiological interaction (PPI) analysis is a method to estimate task-dependent functional connectivity among brain regions [31,32]. The PPI analysis was conducted to specifically address the hypothesis of increased interhemispheric interaction between both MI hand areas during both MVF conditions as a modulating factor of the recruitment of MI_ipsi_ corresponding to the non-mirrored hand as proposed in prior literature [13]. For that purpose, the deconvolved voxel time courses of each subject and session were extracted from the native space coordinates of peak voxels within the contralateral MI (MI_contra_) as revealed by the *t*-contrasts of the first-level analyses of both MVF conditions. We chose MI_contra_ because it was consistently activated in all subjects during both MVF. The fMRI time course of each selected region of interest (ROI) was obtained by using the first eigenvariate of a radial sphere of 5 mm surrounding each peak voxel. Based on the individual voxel time series, statistical parametric maps for each subject and MVF condition were created, representing regions in which the fMRI signal was predicted by the PPI term (the cross product of the physiological and the psychological factors) [31]. Both the physiological and psychological factors were included in the design matrix as confounding variables. Furthermore, we include the white matter- and cerebrospinal fluid-signal as nuisance regressors [32].

The first-level (session) statistics were entered into a second-level group statistic to reveal task-dependent functional connectivity for both MVF conditions. *Z* (Gaussianized T) statistic images were thresholded using a cluster-based threshold of *z* > 3.0 and a whole-brain corrected cluster significance threshold of p = 0.05.

### Analysis of subjective ratings

The seven-point-ratings on the intensity and vividness of mirror illusions during both MVF conditions were analyzed by SPSS Statistics 20.0.0 software (IBM Corporation, New York, USA). Comparisons of the two mirror illusions items between conditions were conducted using paired sample *t*-tests with Bonferroni adjusted alpha-levels of 0.025 (0.05/2).

## Results

### Ratings on mirror illusions

The participants did not report any problems in performing either of the MVF tasks and showed high compliance with both MVF devices. We did not find significant differences in the assessed items between the conditions (vividness: *t*_19_ = 0.18, *p* = .86; intensity: *t*_19_ = 0.2, *p* = 0.84). The mean values of the ratings for the items used in both conditions were between 4.95 and 5.8 (Table 1).

**Table 1:** Ratings on the intensity and vividness of mirror illusions for the mirror box and mirror glasses conditions.

Mirror illusion item	Mirror glasses	Mirror box	*t* (19)	*p*
Intensity (M+SD)	5.8 (± 1.44)	5.75 (± 1.68)	0.2	0.84
Vividness (M+SD)	5.3 (± 1.59)	4.95 (± 2.11)	0.18	0.86

Results are reported with Mean ± Standard Deviation of the Mean (*M* ± *SD*). Comparisons of the two items between conditions were conducted with paired sample *t*-tests with Bonferroni adjusted alpha-values of 0.025 (0.05/2). Numerical rating scale ranging from 1 (‘as clear and vivid as a real perceptual experience’) to 7 (‘not at all clear and vivid’).

### Functional Imaging Data

#### Task-related brain activation in both MVF conditions

**Fig 2:**
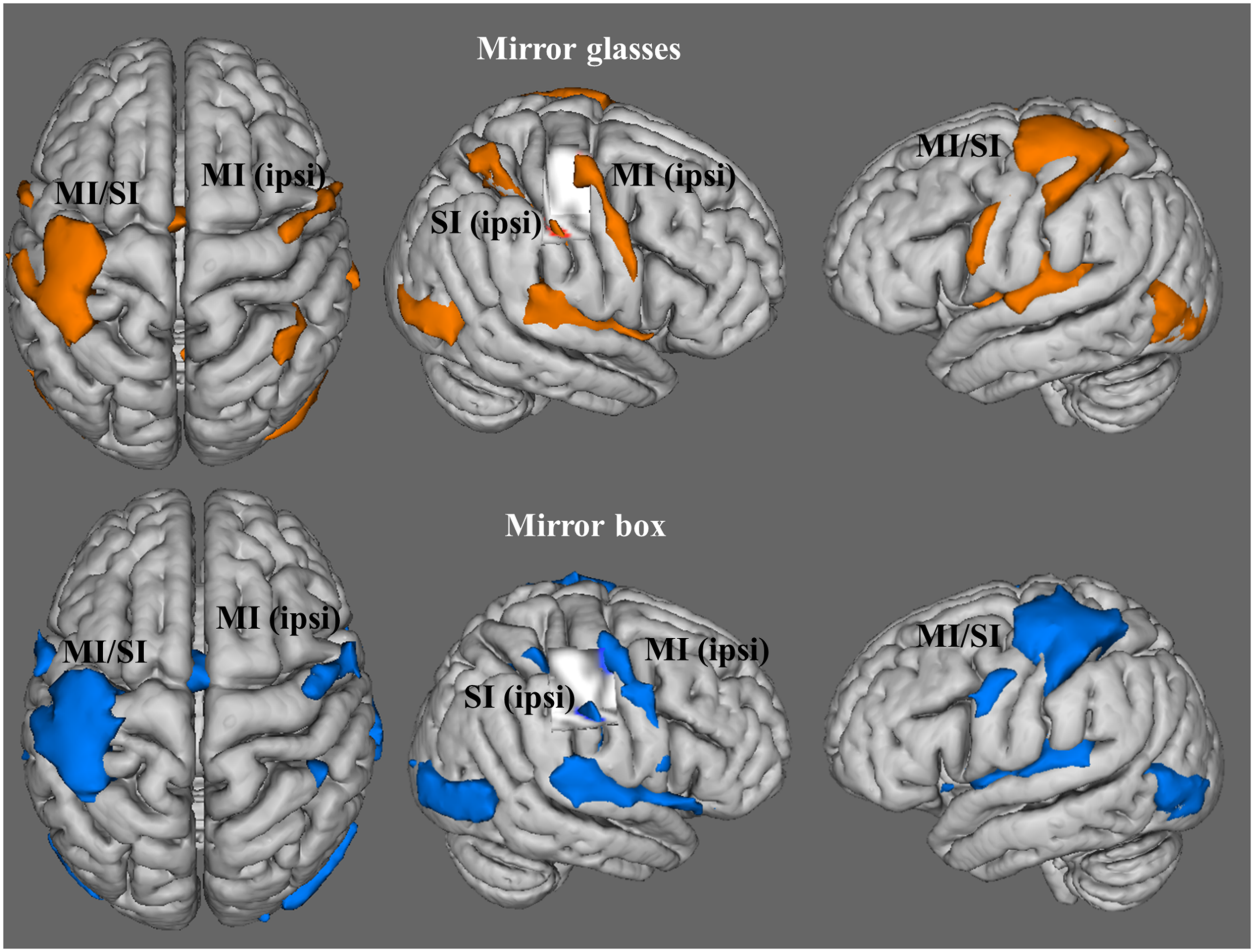
Task-related brain activation for the mirror glasses and mirror box conditions. fMRI activations were mapped on a FSL render image. MI/SI = primary motor/somatosensory cortex, ipsi = ipsilateral to the executing (right) hand.

Imaging data revealed significant fMRI activations in the left sensorimotor cortex corresponding to the moving right hand in both MVF conditions (MNI coordinates: MB x = -40, y = -22, z = 56, *Z* = 7.0; MG x = -38, y = -24, z = 60, *Z* = 7.26). Additionally, a significant cluster of activation was found in the right sensorimotor cortex representing the non-mirrored left hand in both MVF conditions (MB x = 40, y = -36, z = 52, *Z* = 3.64; MG x = 42, y = -12, z = 62, *Z* = 4.99) (Table 2, Fig 2). Furthermore, significant clusters of activation were found in the supplementary motor area (SMA), the premotor cortex (PMC), the ipsilateral cerebellum and the secondary somatosensory cortex (SII). Besides these sensorimotor activations, we found additional peak voxels in the primary auditory cortex (Heschl’s gyrus) and visual areas like the lateral occipital cortex (LOC) (Table 2, Fig 2).

**Table 2:** Brain regions and peak voxel coordinates showing significant task-related brain activation for the mirror box and mirror glasses conditions.

Region: left hemisphere, contralateral to the moving hand	MNI-coordinates			*z*-score	Extent [voxels]	Region: right hemisphere, ipsilateral to the moving hand	MNI-coordinates			*z*-score	Extent [voxels]
	x	y	z				x	y	z		
**Mirror glasses**											
Precentral gyrus	-60	6	30	4.53	148	Precentral gyrus	56	0	52	5.48	838
Precentral gyrus	-34	-22	70	6.47	5362	Precentral gyrus	42	-12	62	4.99	838
Postcentral gyrus	-38	-24	60	7.26	5362	Postcentral gyrus	54	-18	40	4.24	107
Postcentral gyrus	-42	-26	50	6.71	5362	Superior parietal lobule	38	-48	70	4.13	351
Supplementary motor area	-4	-6	58	4.89	258	Planum temporale	60	-16	8	5.94	1547
Putamen	-26	-8	12	4.33	146	Cerebellum	8	-56	-10	5.9	115
Lateral occipital cortex	-44	-76	4	5.79	1988	Lateral occipital cortex	50	-64	6	6.02	1548
						Lateral occipital cortex	30	-78	32	4.31	115
**Mirror box**											
Precentral gyrus	-62	2	32	4.6	204	Postcentral gyrus	40	-36	52	3.64	92
Precentral gyrus	-40	-22	56	7	5967	Secondary somatosensory cortex	66	-20	18	6.43	3515
Postcentral gyrus	-38	-24	62	6.97	5967	Cerebellum	8	-58	-10	5.54	109
Heschl's gyrus	-50	-20	8	7.08	5967	Lateral occipital cortex	48	-68	-2	6.41	1701
Supplementary motor area	-4	-4	60	5.7	445	Occipital pole	16	-96	-8	3.98	97
Thalamus	-14	-20	2	5.4	109						
Lateral occipital cortex	-48	-76	6	5.1	1827						
Occipital fusiform gyrus	-18	-80	-10	4.3	181						

Areas of significant fMRI-response were determined using clusters identified by a *z* > 3.0 threshold and a corrected cluster threshold of p = 0.05 assuming a Gaussian random field for the *z*-statistics. Coordinates are displayed in the Montreal Neurological Institute (MNI152) space.

The direct comparisons between both MVF conditions (MG > MB and MG < MB) yielded no significant differences in whole-brain activations, indicating comparable patterns of fMRI activations for both MVF tasks.

#### Task-dependent functional connectivity between hand areas during both MVF conditions

**Fig 3:**
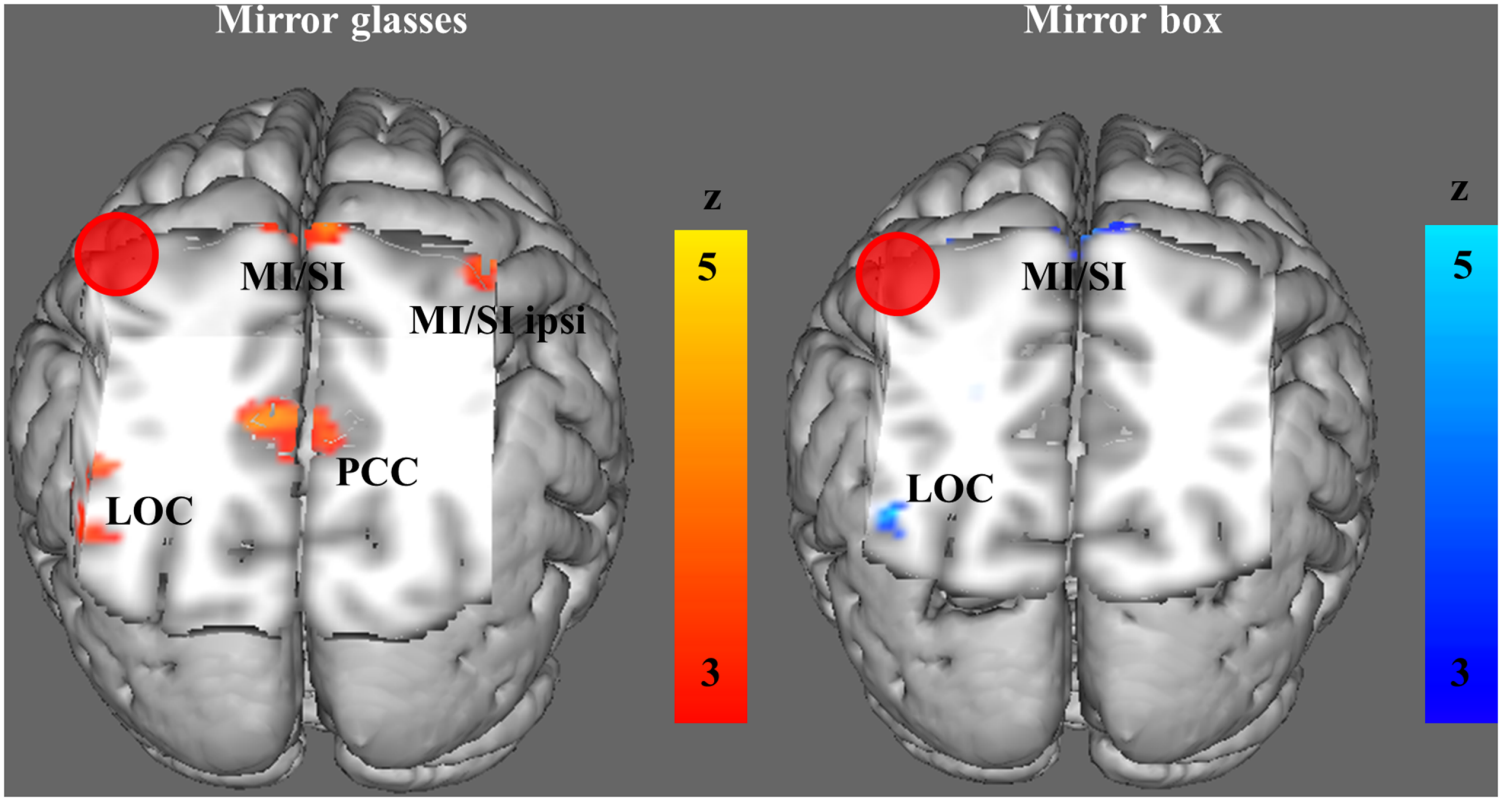
Brain regions showing significant positive psychophysiological interactions (PPI) with the motor representation of the moving hand. fMRI activations were mapped on a FSL render image. For illustrative purposes the spherical seed region of interest in the left primary motor cortex is also shown as red-colored sphere. MI/SI = primary motor/somatosensory cortex, LOC = lateral occipital cortex, PCC = posterior cingulate cortex, ipsi = ipsilateral to the executing (right) hand.

In order to test whether the motor representation of the actually moving hand (MI_contra_) was functionally coupled with MI_ipsi_ of the non-mirrored (hidden) hand, we used a PPI analysis with a seed region in MI_contra_. We found a significant positive psychophysiological interaction between MI_contra_ with the sensorimotor representation of the non-mirrored hand (x = 40, y = -24, z = 66, *Z* = 3.91) in the MG condition (Table 3, Fig 3). No significant positive correlation was found between MI_contra_ and the sensorimotor representation of the non-mirrored hand in the MB condition. In both MVF conditions, MI_contra_ showed significant positive functional connectivity with frontal lobe regions (middle and superior frontal gyrus) and the LOC. Furthermore, in both MVF conditions significant positive psychophysiological interactions were found with the precentral gyrus. However, these peak voxels were located too medially to be a correlate of the mirrored right hand (MB x = 2, y = -26, z = 78; MG x = 6, y = -28, z = 76) (Table 3, Fig 3). In the MB condition we found further positive psychophysiological interactions with the SMA. In the MG condition we found additionally significant task-related functional connectivity with the middle and superior frontal gyrus, the paracingulate gyrus, the angular gyrus and the posterior cingulate gyrus (Table 3, Fig 3).

**Table 3:** Brain regions showing significant positive psychophysiological interactions (PPI) with the motor representation of the moving hand for the mirror box and mirror glasses conditions.

Region: left hemisphere, contralateral to the moving hand	MNI-coordinates			*z*-score	Extent [voxels]	Region: right hemisphere, ipsilateral to the moving hand	MNI-coordinates			*z*-score	Extent [voxels]
	x	y	z				x	y	z		
**Mirror glasses**											
Superior frontal gyrus	-22	30	46	4.81	671	Precentral gyrus	6	-28	76	4.05	249
Middle frontal gyrus	-38	10	50	4.16	156	Postcentral gyrus	40	-24	66	3.91	95
Posterior cingulate gyrus	-10	-44	34	4.01	246	Paracingulate gyrus	2	40	-12	4.14	200
Angular gyrus	-46	-56	30	3.94	135						
Lateral occipital cortex	-34	-74	42	3.93	203						
**Mirror box**											
Precentral gyrus	-28	-26	74	3.86	77	Precentral gyrus	2	-26	78	3.79	172
Middle frontal gyrus	-26	32	46	3.84	282						
Supplementary motor area	-2	-2	74	3.66	78						
Lateral occipital cortex	-40	-70	34	3.86	111						

Seed regions of interests derived from subject specific peak voxels in the primary motor cortex of the single contrasts mirror glasses and mirror box. PPIs were calculated based on deconvolved fMRI signals from individual seed voxels obtained with a radial sphere of 5 mm. Areas of significant fMRI-responses were determined using clusters identified by a z > 3.0 threshold and a corrected cluster threshold of p = 0.05 assuming a Gaussian random field for the z-statistics. Coordinates are displayed in the Montreal Neurological Institute (MNI152) space.

We also tested for significant negative psychophysiological interactions (decouplings). We did not find significant decouplings between the representations of both hands in the predefined ROIs in either of the MVF conditions. For an overview about significant negative psychophysiological interactions other than those in the specified ROIs see S1 Table.

## Discussion

The present study evaluated the utility of MG by comparing it with the MB and yielded three important results: (1) We did not find significant differences in subjective ratings capturing mirror illusion capacity between either MVF intervention, indicating similar capabilities of both to induce mirror illusions. (2) We found similar patterns of task-related brain activation for both conditions, including the sensorimotor representation of the non-mirrored hand as well as other brain areas typically found in MVF tasks [13,33]. Critically, the direct comparison of both MVF interventions yielded no significant differences in fMRI activation. (3) Furthermore, we found increased interhemispheric connectivity between both hand representations only in the MG condition. This suggests that the motor representation of the non-mirrored hand in the MG condition is modulated via this interhemispheric connection. Due to the fact that the hand region in MI_ipsi_ was activated in both MVF conditions we assume that the MB condition works by a different neural mechanism.

### Comparable subjective quality of mirror illusions

To our knowledge this is the first study quantifying the subjective quality of MG in comparison to the well-established MB. The MG have been discussed to be superior to the classical MB and even virtual-reality applications of the MB because they provide a naturalistic view on the reflection of the actually moving limb without seeing the mirrored limb additionally which potentially has a distracting effect [18–20]. Neither the vividness nor the intensity of mirror illusions differed significantly between both mirror tasks. The most notable difference between both MVF conditions was the presentation of only the visual reflection of the moving right hand in the MG compared with both hands appearing to move in synchrony in the MB condition. We hypothesized to find higher subjective ratings on mirror illusions in the MG condition, because it has been proposed that seeing the moving hand in addition to the visual reflection might interfere with mirror illusions and the accompanying recruitment of the sensorimotor representation of the hidden hand [18,19]. Despite of the low to medium high ratings for the mirror illusion items used, the subjective ratings were comparable to other studies using these items [15,16] including patient studies demonstrating a therapeutic effect of MVF [3]. It is important to note that we did not instruct the participants to perform motor imagery during the MVF task. We used the standard (original) instruction for clinical studies as has been used, for example, by Ramachandran & Rogers-Ramachandran [34], who originally reported the effects of mirror training on phantom pain. It has been shown that mirror illusions and the concomitant recruitment of the affected limb representation can be improved by combining MVF with motor imagery [9,35]. Thus, we assume that the moderate levels of induced mirror illusions can be increased when MVF is combined with motor imagery.

### Comparable task-related brain activation

We found comparable patterns of functional brain activation between both MVF conditions, including those areas that have been shown to be typically activated in a motor MVF task [13,33]. In contrast to our hypothesis, we did not find significant differences in fMRI activations in the MI_ipsi_ corresponding to the hidden left hand or in any other brain region between both MVF tasks [19].

The visual illusion of the moving hand has been discussed to be the experimental substrate of MVF-related excitation of the MI corresponding to the non-mirrored hand [19]. In both MVF conditions, we found extended fMRI activations in the right sensorimotor cortex, corresponding to the non-mirrored (hidden) hand, in addition to a significant activation of the sensorimotor representation of the actually moving hand. A recruitment of the sensorimotor representation ipsilateral to the moving hand during a MB task was also found in former fMRI studies using MVF [15,16,36] and has been reported to be a stable neuronal correlate in a recent meta-analysis including 33 MVF studies [13]. It has been shown that ipsilateral hand movement [37,38] as well as passive observation of contralateral limb movements can induce excitability changes in MI_ipsi_ [39,40]. The interaction between ipsilateral motor observation (as realized in a MB task) and contralateral motor execution has been discussed to drive the excitability changes in MI_ipsi_ during MVF [41]. Garry et al. [41] were able to show that the motor observation component alone increases excitability in MI_ipsi_, whereby facilitation of MI_ipsi_ excitability was strongest with the mirror reflection. Moreover, Diers et al. [15] found increased fMRI activation in MI_ipsi_ in a group of healthy controls and amputees without phantom limb pain in a motor execution as well as a MVF task, but activity was higher with MVF, which suggest an additional effect of the motor observation component for the recruitment of the hand representation corresponding to the hand seen in the mirror. We did not include a pure motor execution condition in this study, but we can conclude from results of previous studies that activations would be located in similar regions, although less prominent [15,24,42].

In a magnetoencephalographic study, Hadoush et al. [19] investigated the effects of seeing the physically moving hand in addition to the mirror reflection of the moving hand on MI_ipsi_ excitability within a classical MB setup. The subjects were tested in a within-subjects design performing a MB task with either their actually moving hand out of view or visible. Hadoush et al. [19] reported a higher capability to recruit MI_ipsi_ and a clearer visual illusion when the executing hand was out of view. We also hypothesized to find a stronger recruitment of MI_ipsi_ in the MG condition because subjects can more easily focus on the mirror illusion [18]. Although we did not use an additional item to specifically assess the potentially distracting effect of seeing the executing hand on mirror illusions in the MB condition [19], we found no significant differences in the capability to recruit the MI_ipsi_ between the two MVF conditions as revealed by the direct comparison between them. In contrast to Hadoush et al. [19], we did not instruct the subjects to perform motor imagery during the MVF task. It has been discussed that mirror illusions and the concomitant recruitment of the affected limb representation can be improved by combining MVF with motor imagery [9,13,35] and possibly the additional effect of motor imagery might differ between the MB and MG condition by seeing just one compared with two hand moving in synchrony. Thus, the proposed beneficial effect on MI_ipsi_ recruitment caused by disabling the vision of the actually moving limb compared with seeing both hands moving in synchrony cannot be supported by our findings.

Moreover, we found additional fMRI activations during the mirror tasks in PMC, the ipsilateral cerebellum, SMA, the thalamus, the LOC as well as SII, which constitute brain regions typically activated in hand motor tasks like the MB task [13,33]. Clusters of activation were further found in the primary auditory cortex, which were expected due to the auditory pacing signal present during the movement trials in both mirror tasks.

Despite the differences in the amount of visual input between both MVF conditions by seeing just one hand in the MG compared with two hands appearing to move in synchrony in the MB, neither the single condition contrasts nor the direct comparison between both MVF conditions revealed significant differences in visual areas. In both MVF conditions clusters of activation in the LOC showed similar cluster extensions and peak maxima between both hemispheres.

### Different patterns of task-dependent functional connectivity

It has been proposed that the MVF related recruitment of the affected motor limb representation (MI_ipsi_) is due to contralateral projections arising from the motor representation of the moving (intact) limb (MI_contra_) [3,43,44]. To specifically address this hypothesis of an MVF-related increase in interhemispheric connectivity between both motor hand representations, we applied PPI analysis with individually defined ROIs in the MI_contra_ [13]. So far there is a lack of studies on functional connectivity between brain areas to reveal the neuronal mechanisms underlying MFV [13].

We found a significant increase in interhemispheric connectivity between MI_contra_ and the sensorimotor representation of the non-mirrored hand in the MG condition, but not in the MB condition. The absence of significant interhemispheric communication in the MB condition is in line with the finding of a recent MVF study examining motor improvement in the limb seen in the mirror in two patients with callosal disconnection [45]. These callosal patients showed improved motor function in the untrained hand seen in the mirror after mirror training, which cannot be explained by intermanual transfer mediated by transcallosal fibers in these subjects. Moreover, Hamzei et al. [44] found increased functional and effective connectivity between various brain regions, but not between both motor hand areas in a group of healthy volunteers performing mirror training. Thus, the recruitment of the sensorimotor representation corresponding to the non-mirrored hand was likely not mediated by interhemispheric communication via transcallosal fibers between the hand areas in the MB condition.

We found a significant increase in task-related interhemispheric connectivity only in the MG condition. However, in both MFV conditions the ipsilateral sensorimotor representation of the non-mirrored hand was significantly activated and fMRI activity did not differ between both MVF conditions as revealed by the direct comparison between both MVF conditions. Thus, our findings indicate that the mechanism, by which the ipsilateral sensorimotor representation of the non-mirrored hand was recruited, might vary between both MVF conditions. Whereas interhemispheric communication seems to be important for the recruitment of MI_ipsi_ in the MG condition, it might just play a minor role in the MB condition. How can this difference in the recruitment of the sensorimotor representation of the non-mirrored hand be explained?

Our ROI was located in the motor representation of the moving hand, in order to specifically address the hypothesis of increased interhemispheric communication mediating the recruitment of MI_ipsi_. Thus, we can only speculate which alternative mechanism might account for the recruitment of sensorimotor representation of the hidden hand in the MB. It has already been proposed that afferent information from the visual cortex might re-establish coherence in the limb representation in MI_ipsi_ by recruiting the preserved motor representation in patient groups [46]. In both MVF conditions we found increased psychophysiological interactions between the LOC and MI_contra_, indicating that afferent input from visual areas might be an attractive candidate for the recruitment of the sensorimotor representation of the non-mirrored hand.

### Study limitations

A limitation of the current study is that we only looked at the instant neuromodulatory effects of MVF. Thus, we cannot exclude the possibility of use-dependent dynamics in functional brain activity by long-term training with our MVF devices [13,44].

Furthermore, it has to be considered that healthy subjects performed both mirror tasks. In future studies, the MG will have to be evaluated in specific patient groups such as patients with specific motor deficits or chronic pain.

A further limitation of this study is that we did not apply measures of effective connectivity (e.g. dynamic causal modelling or Granger causality) because our experimental design was not factorial and therefore is not suitable for applying effective connectivity analysis [47,48]. As highlighted in the original publication on dynamic causal modelling by Friston et al. [49] a multi-factorial design with one factor assumed to be a driving input (e.g. sensory stimulation) and another factor acting as modulatory input (e.g. attention) is suggested.

## Conclusions

Based on comparable patterns of brain activation and subjective ratings on mirror illusions, we conclude that MG might be a versatile substitute of the MB in the treatment of chronic pain as well as the functional recovery in different patient groups. Compared with the MB, MG might be favoured due to their higher manageability in everyday therapy and research.

Moreover, we found evidence that the recruitment of the hand representation of the non-mirrored hand might be mediated by interhemispheric communication in the MG but not in the MB condition, indicating that different neural mechanisms might contribute to the recruitment of the cortical hand representation of the non-mirrored hand in the MB versus MG condition. This difference might be explained by the conceptual difference of seeing both hands moving in synchrony (MB) versus seeing only the visual reflection of the moving hand (MG).

## Supporting Information

S1 Table Brain regions revealing significant negative psychophysiological interactions (PPI) with the motor representation of the moving hand for the mirror box and mirror glasses conditions. Areas of significant fMRI-responses were determined using clusters identified by a *z* > 3.0 threshold and a corrected cluster threshold of p = 0.05 assuming a Gaussian random field for the *z*-statistics. Coordinates are displayed in the Montreal Neurological Institute (MNI152) space.
(PDF)
