# Supplementary material for: Do Mirror Glasses Have the Same Effect on Brain Activity as a Mirror Box? Evidence from a Functional Magnetic Resonance Imaging Study with Healthy Subjects
Source: PLoS One. 2015 May 27;10(5):e0127694. doi: 10.1371/journal.pone.0127694 (PMC4446290; doi:10.1371/journal.pone.0127694)
Supplement: S1 Table — Areas of significant fMRI-responses were determined using clusters identified by a z > 3.0 threshold and a corrected cluster threshold of p = 0.05 assuming a Gaussian random field for the z-statistics. Coordinates are displayed in the Montreal Neurological Institute (MNI152) space. (PDF) [file pone.0127694.s001.pdf]

S1 Table. Brain regions revealing significant negative psychophysiological interactions (PPI) with the motor representation of the moving hand for the mirror box and mirror glasses conditions.

Region:<br>left hemisphere (contrateral to the moving hand)	MNI-coordinates x	y	z	*z*-score	extent [voxels]	Region:<br>right hemisphere (ipsilateral to the moving hand)	MNI-coordinates x	y	z	*z*-score	extent [voxels]
**Mirror glasses**											
Secondary somatosensory cortex	-56	-38	22	4.89	2143	Inferior frontal gyrus	48	10	18	3.92	90
Thalamus	-8	-16	4	3.92	73	Anterior cingulate cortex	6	12	38	4.24	152
						Central opercular cortex	44	8	2	4.57	1026
						Posterior cingulate cortex	8	-26	44	4.07	71
						Supramarginal gyrus	60	-40	18	5.24	1175
**Mirror box**											
Anterior cingulate cortex	-8	10	38	4.19	305	Insular cortex	40	10	-4	4.71	1195
Insular cortex	-34	22	-2	4.85	1154	Superior temporal gyrus	62	-36	10	4.77	1681
Planum temporale	-52	-24	4	4.36	905						

Areas of significant fMRI-responses were determined using clusters identified by a $z > 3.0$ threshold and a corrected cluster threshold of $p = 0.05$ assuming a Gaussian random field for the *z*-statistics. Coordinates are displayed in the Montreal Neurological Institute (MNI152) space.
